# Low dose quetiapine induced galactorrhoea: a case report

**DOI:** 10.1186/1745-0179-3-12

**Published:** 2007-07-24

**Authors:** Maneesh Gupta

**Affiliations:** 1 Josiah Thomas Memorial Hall, Cornwall Partnership NHS Trust, Camborne, Cornwall, TR14 8LQ, UK

## Abstract

**Background:**

Quetiapine causes less prolactin elevation and/or galactorrhoea than other atypical antipsychotics.

**Case Presentation:**

Ms AB had galactorrhoea and raised prolactin levels at only 100 mg of quetiapine daily.

**Conclusion:**

Low dose quetiapine can also cause galactorrhoea.

## Background

Prolactin elevation and consequent galactorrhoea is a known side effect of antipsychotics [1]. Under normal circumstances, dopamine-producing neurons in the arcuate nucleus of the hypothalamus release dopamine in the hypothalamo-hypophysial blood vessels of the median eminence, which supply the pituitary gland. This dopamine inhibits prolactin secretion. When antipsychotics are taken, dopamine receptor blockade in the tuberoinfundibular pathway [2] releases the lactotrope cells in the pituitary, which secrete prolactin, leading to consequent hyperprolactinemia. This in turn can cause side effects like galactorrhoea, breast enlargement, menstrual disturbances, amenorrhoea or sexual disturbances.

Galactorrhoea is a troublesome and embarrassing side effect of antipsychotics. Quetiapine is the least galactogenic antipsychotic and has been recommended as the treatment to use if a patient has galactorrhoea on haloperidol [3]. It has been shown to have little effect on prolactin levels [4,5]. One report [6] mentioned substituting quetiapine for olanzapine in a client who had prolactinemia and consequent galactorrhoea. Quetiapine was used in a 400 mg daily dose and both galactorrhoea and prolactinemia subsided. This was in keeping with evidence that clozapine and quetiapine are the least likely to cause prolactinemia among the atypical antipsychotics [7].

Keller et al [5] describe a series of five cases where they switched to quetiapine following prolactinemia on other atypicals and had no recurrence of endocrinological symptoms. The E-SOHO [8] study reported lower incidence of side effects related to prolactin elevation in those treated with quetiapine, clozapine and olanzapine. A randomized study in Turkey [9] showed that cohorts on quetiapine had no prolactin elevation while two of those on haloperidol had galactorrhoea.

One case report of galactorrhoea associated with quetiapine [10], focused on the role of venlafaxine and its action on dopamine in the brain.

Unlikely thus that it is, this case report discusses galactorrhoea in a client on a very low dose of quetiapine.

## Case Presentation

Ms AB, a 31 year old lady known to be suffering from personality disorder complained of troublesome voices off and on for the last few years. More recently she felt that these voices were telling her to self-harm. These were presumed to be dissociative in nature and no antipsychotics had been prescribed consistently. We added quetiapine 50 mg at night with a plan to increase the dose gradually. However, an increase to 100 mg caused drowsiness and the patient felt unable to increase the dose further. She also complained of muscle spasms which improved on procyclidine. Ten days after the increase to 100 mg, the client complained of a feeling of heaviness in her breasts and later of galactorrhoea. A serum prolactin estimation (17 days on 100 mg) showed raised levels (603 mu/l; N = 102–496). A retrospective medicine chart review showed only two extra doses of 50 mg having been used in the last week before the estimation.

I investigated a number of other potential reasons for elevation of prolactin and consequent galactorrhoea. Thyroid stimulating hormone (TSH) level was within normal limits. A brain scan was not performed, as the elevation was less than two times the upper limit of normal, which made it unlikely to be a pituitary tumour. Other medications that the client was on included divalproex sodium 1250 mg, gabapentin 900 mg and venlafaxine 150 mg. These were on similar doses before the quetiapine was started and after the quetiapine was stopped. At the time of the second prolactin estimation, these were still continuing with no symptoms and the prolactin levels were normal.

The quetiapine dose was reduced to 50 mg and then stopped. The galactorrhoea subsided in three days and the heaviness in breasts remitted in seven days.

A repeat serum prolactin estimation two months after stopping the quetiapine showed normal levels (359 mu/l; N = 102–496). No other antipsychotic was tried.

## Discussion

Atypical antipsychotics cause less prolactin elevation and/or galactorrhoea than typical antipsychotics [2]. Quetiapine is an atypical antipsychotic that has been shown to have transiently high dopamine (D2) receptor occupancy [11]. This is explained as the reason for its low incidence of extrapyramidal (EPS) and prolactin elevation. In 2002, Kapur and scientists at Janssen Pharmaceuticals, published results indicating that quetiapine differentially occupied striatal and pituitary D2 receptors [12]. This explained the less frequent association of quetiapine with prolactin elevation. No wonder then that quetiapine is deemed to be the least galactogenic of all atypical antipsychotics.

Yet, there have been some reports of its adverse effect on the prolactin-galactorrhoea mechanism. Alexiadis et al, in a letter, mention elevation of prolactin due to quetiapine within 90 minutes [13]. However, it did not report any consequent galactorrhoea. Pae et al present a report of quetiapine and venlafaxine leading to galactorrhoea [10]. They focus on the role of venlafaxine on dopamine and the consequent galactorrhoea. In this case report, though the client was on venlafaxine along with quetiapine, galactorrhoea subsided on stopping quetiapine, while venlafaxine continued.

An adolescent male has been reported to suffer from galactorrhoea on standard doses of quetiapine, which subsided on reducing the dose to 400 mg/day [14]. The report discusses the proven lack of evidence of symptomatic endocrinological symptoms in adolescents with quetiapine; and cautions that this may "rarely be encountered". It is interesting that galactorrhoea appeared after three months of maintenance treatment and on 600 mg/day, whereas in Ms AB it appeared within ten days and on a low dose of only 100 mg/day.

While evidence suggests otherwise, galactorrhoea with quetiapine may be an uncommon side effect, which needs to be ruled out in a symptomatic patient. Clinically, emergent galactorrhoea is likely to be an embarrassing and distressing iatrogenic side effect of any treatment. Hyperprolactinemia, on the other hand may not be apparent and may not lead to any dysfunction in the patient. Quetiapine's use is acceptable when galactorrhoea does not emerge, and patients' are likely to accept it even if prolactin is elevated.

## Conclusion

Quetiapine, an atypical antipsychotic, may cause galactorrhoea due to prolactin level elevation. In this client this effect appeared at a very low dose of just 100 mg a day within ten days. Stopping the antipsychotic, relieved galactorrhoea and normalised the prolactin levels.

This case report adds to our knowledge of side effects due to quetiapine. Clinicians may want to look at all antipsychotics, including quetiapine, as possible reasons for galactorrhoea. However, it must be realised that this is a case report and the actual incidence might be less common.

## Competing interests

The author has accepted hospitality from various pharmaceutical companies in UK. He has accepted honoraria to speak at clinical gatherings from Astra Zeneca and Wyeth Laboratories. The author holds some shares in some Indian pharmaceutical companies.
